# Migration of Chlorine in Plant–Soil–Leaching System and Its Effects on the Yield and Fruit Quality of Sweet Orange

**DOI:** 10.3389/fpls.2021.744843

**Published:** 2021-10-11

**Authors:** Xiaodong Liu, Chengxiao Hu, Zongying Zhu, Muhammad Riaz, Xiaoman Liu, Zhihao Dong, Yu Liu, Songwei Wu, Zhenhua Tan, Qiling Tan

**Affiliations:** ^1^Micro-Element Research Center/Hubei Provincial Engineering Laboratory for New Fertilizers, College of Resources and Environment, Huazhong Agricultural University, Wuhan, China; ^2^Key Laboratory of Arable Land Conservation (Middle and Lower Reaches of Yangtze River), Ministry of Agriculture, Huazhong Agricultural University, Wuhan, China; ^3^Root Biology Center, College of Natural Resources and Environment, South China Agricultural University, Guangzhou, China; ^4^Xiangnan Navel Orange Comprehensive Test Station, Hunan, China

**Keywords:** chlorine-containing fertilizer, chlorine migration, leaf nutrients, citrus yield, fruit quality

## Abstract

Chlorine (Cl) is indispensable for the growth of plants. While rarely systematic reports are available for the effect of Cl-containing fertilizers on citrus production. This study aimed to investigate the impacts of various Cl-containing fertilizers on the nutrients in the leaves, the yield and quality of sweet orange, and the Cl migration in the plant–soil–leaching system. A 5-year field experiment (2016–2020) with five Cl treatments (0, 75, 150, 450, and 900 kg ha^−1^), and soil core lysimeter test with five Cl levels (0, 150, 225, 300, and 450 kg ha^−1^) were carried out. The results showed that 77.0% of Cl leached into above 60 cm deeper soil layer, with calcium as the main accompanying ions, resulting in less Cl being absorbed by the citrus plants. The content of Cl in the leaves and soil was enhanced by the increasing input of Cl-containing fertilizer, without yearly increased characteristics, under a mean annual rainfall of 1,474 mm. Chlorine significantly increased the yield (13.24–37.8 9%), fruit weight, and vitamin C (Vc), in addition to enhancing the flavor and the juice yield of sweet orange via improving the absorption of N and K. Moreover, the long-term application of potassium sulfate has elevated the accumulation of sulfur in the soil and in leaves; it is becoming a potential risk factor for citrus production. Taken together, the application of Cl-containing fertilizer in sweet orange is feasible, and trace absorbance of Cl could improve the yield and fruit quality of sweet orange.

## Highlights

- About 77.0% of Cl leached into deep soil and was mainly accompanied by Ca^2+^.
- Cl increased the yield, fruit weight, and Vc in sweet orange.
- Cl improved the flavor and juice yield of citrus *via* enhanced N and K content.
- Sulfur accumulated in the soil and leaves might be a risk factor for citrus production.

## Introduction

Chlorine (Cl) is deemed as an essential micronutrient in plants and participates in a variety of physicochemical processes (Raven, 2017; Colmenero et al., 2019). The Cl requirement is relatively low and is usually met by air, rainfall, soil, and potassium fertilizer (Wang et al., 2020). The content of Cl in most plants is observed in the range of 2–20 g kg^−1^ (Broadley et al., 2012; Colmenero et al., 2019). Chloride primarily accumulates in the vacuoles, reaching concentrations of up to 150 mM, and can serve as a permeable substance to drive water flow (Geilfus, 2018b). Furthermore, the accumulation of Cl could improve the utilization of nitrogen (N) by decreasing the compartmentalization of nitrate (N-NO_3_) in the vacuole and promoting its assimilation. In the previous study, it was observed that the utilization of N increased by 0–22% in citrus and olive, and increased by 60–80% in tomato and tobacco when aided by Cl (Rosales et al., 2020).

Chlorine also appears as a toxic element for some specific plants, such as citrus, tobacco, and grapevine, which are defined as Cl sensitive crops (Xu et al., 1999). Long-term use of river water with a salt level of 2.5 dS m^−1^ for irrigation caused the soil salinity to increase linearly;, the citrus yield reduced by 17%, and the trunk-diameter growth slowed down 59% yearly (Prior et al., 2007). The adverse environment and the mismanagement (e.g., saline soils, lower rainfall, excessive fertilizer, and irrigation of saline water) mean it is easy to cause Cl poisoning and stunt plant growth. Farmers have historically failed to define the safe dose of Cl-containing fertilizer for this Cl-sensitive crop. Therefore, citrus production in China has always been based on the use of potassium sulfate (K_2_SO_4_) as K fertilizer, instead of potassium chloride (KCl). The long-term application of K_2_SO_4_ has witnessed the sharp rise in soil sulfate (${\text{SO}}_{4}^{2-}$) (Szynkiewicz et al., 2011), leading to strong acidification of the environment and the leaching of ${\text{SO}}_{4}^{2-}$ resulting in an excess of ${\text{SO}}_{4}^{2-}$ in the soil (Tabak et al., 2020). Consistently, previous research has increased concerns that further input of sulfur could increase the risk of methylmercury (MeHg) production (a bio-accumulative neurotoxicant), which may enhance the dietary MeHg exposure (Lei et al., 2021). Additionally, excessive sulfur (S) greatly increased the accumulation of cadmium in roots, resulting in a diminished rice yield (Fan et al., 2010). The reckless pursuit of yield leads to the problem of excessive fertilization in most of the citrus-producing areas in China (Liu et al., 2021). Thus, the long-term application of K_2_SO_4_ caused the increasing S content in the leaves and soil of ‘Guanximiyou’ pomelo orchards, which exacerbated the acidification of soil and the leaching of calcium (Ca) and Mg (magnesium). Therefore, it is essential to reduce the K_2_SO_4_ application in production (Lin et al., 2020).

Citrus, an indispensable economic crop, accounts for one-quarter of the total fruit yield in the world (Qi and Qi, 2016), and it is mainly planted in acid soil. At present, excessive application of N, phosphorus (P), and K fertilizer leads to increasingly serious acidification of soil in citrus orchards, which inhibits nitrification and promotes N loss (Quaggio et al., 2014). Soil acidification enhancement has releases other cations (e.g., Ca, K, Mg, Al, Fe), and led to vast amounts of ${\text{SO}}_{4}^{2-}$ absorbed into soil (Souza et al., 2006), which may advance Cl leaching. The problem of planting citrus in acid soil has become increasingly prominent. It is quite necessary to conduct deep-reaching research and comprehensive analyses of cation and anion movement in acid soil. Therefore, acid soil was selected for this study aiming to investigate the migration characteristics of Cl in plant-soil-leachate and analyze the application potential of Cl-containing fertilizer with the employment of the soil core lysimeter experiments (1); and to identify the effect of Cl on the nutrient content in the leaf and the fruit quality of sweet orange (2).

## Materials and Methods

### Experimental Materials and Design

The field experiment was conducted during 2016–2020 on 7-year-old trees of Newhall sweet orange [*C. sinensis (L.) Osbeck*] grafted onto trifoliate orange [*Poncirus trifoliata (L.) Osbeck*] rootstock. The experimental location was at the experimental orchard of the institute of Xiangnan navel orange comprehensive test station, Yizhang, Chenzhou city, Hunan province, China, which is a semi-humid climate. The annual precipitation was 1,850, 1,238, 1,236, 1,742, and 1,309 mm in 2016, 2017, 2018, 2019, and 2020, respectively. Acid red soil (pH = 4.46) was selected in the experiment and the basic properties were assessed (soil organic matter: 28.79 g kg^−1^, available N: 81.71 mg kg^−1^, available P: 74.08 mg kg^−1^, available K: 241.96 mg kg^−1^, and Cl: 10.6 mg kg^−1^). The fertilizer was applied evenly on both sides of trees (on the ditch near the drip line, with a length of 100 cm and a depth of 30 cm). In March, the basic fertilization was applied at 60% of the total fertilization amount, and the remaining 40% was applied as a topdressing in June. Annual application rates of N were 375 kg ha^−1^ as urea, 225 kg ha^−1^ P_2_O_5_ as triple superphosphate, and 225 kg ha^−1^ K_2_O as K_2_SO_4_ or KCl. Considering that excessive potassium would result in stem blight and the decreased citrus yield (Mattos et al., 2003; Quaggio et al., 2011), the additional amount of Cl was provided by CaCl_2_ after KCl application had been completed. There were five levels of Cl in the field experiment, including 0 kg ha^−1^, K_2_O was provided by K_2_SO_4_ (100% K_2_SO_4_); 75 kg ha^−1^, replacing 50% of the amount of K_2_O in K_2_SO_4_ with KCl (50% K_2_SO_4_+ 50% KCl); 150 kg ha^−1^, where K_2_O was all supplied by KCl (100% KCl); 450 kg ha^−1^, K_2_O again completely from KCl and extra Cl was supplied by calcium chloride (CaCl_2_, 300 kg Cl ha^−1^, Cl content is twice that of KCl); and 900 kg ha^−1^, K_2_O again all from KCl with extra Cl supplied by CaCl_2_ (750 kg Cl ha^−1^, Cl content was 5 times that of KCl). The sites were composed of 3 rows with 22 trees of each, and trees were spaced at 4.0 m × 3.0 m (750 trees ha^−1^). During the experimental periods, all the evaluations were carried out on 20 central trees, and the treatments were distributed by completely random block design and were replicated three times with four trees of each.

In order to explore the effect of Cl-containing fertilizer on the ion leaching in leachate, a soil core lysimeter experiment was performed to solve the difficult aquisition of leachate. Considering the size of the soil core lysimeter, the 3-year-old Newhall sweet orange [*C. sinensis (L.) Osbeck*] grafted on trifoliate orange [*Poncirus trifoliata (L.) Osbeck*] was selected for this experiment, which was conducted at Huazhong Agricultural University, Wuhan, China. Therefore, the rate of fertilization has been reduced appropriately. Fertilizer was applied at the total fertilization amount in a circular ditch (30 cm deep and 10 cm wide) near the edge of the lysimeter wall on June 15, 2020. The application rates of N of one season (about 3 months) were 375 kg ha^−1^ as urea, 225 kg ha^−1^ P_2_O_5_ as triple superphosphate, and K_2_O 225 kg ha^−1^ as K_2_SO_4_ or KCl. There were five levels of Cl set up in the soil core lysimeter: 0 kg ha^−1^, K_2_O was from K_2_SO_4_ (100% K_2_SO_4_); 150 kg ha^−1^, K_2_O was all from KCl (100% KCl); 225 kg ha^−1^, K_2_O was all from KCl, supplimented with CaCl_2_ (75 kg Cl ha^−1^, Cl content was half of KCl); 300 kg ha^−1^, K_2_O was KCl and supplimented with CaCl_2_ (150 kg Cl ha^−1^, Cl content was equal to that of KCl) for extra Cl; 450 kg ha^−1^, K_2_O was from KCl and supplimented with CaCl2 (300 kg Cl ha^−1^, Cl content was 2 times that of KCl) for extra Cl. The sites were composed of 2 rows with 15 trees of each, and trees were spaced at an area of 0.3 m^2^ in a soil column (15,000 trees ha^−1^). The treatments were distributed by a completely random block design and were replicated three times with two trees of each. The bulk density of the acid yellow-brown soil (pH = 4.60) was 1.275 g cm^−3^.

The collection of undisturbed soil monolith lysimeters was performed following the rules previously laid out by Cameron et al. (1992) and Zhao et al. (2010). The vertical structure of the design lysimeter used for the large soil core is shown in Supplementary Figure S1. Every lysimeter case consisted of a PVC cylinder (600 mm in internal diameter, 630 mm in external diameter, and 700 mm high). A PVC disk (640 mm in internal diameter, 660 mm in external diameter, and 50 mm thick) was used as the base. Three layers of nylon mesh (diameter 0.074 mm) and acid quartz sand (diameter 0.8 mm, depth of 20 mm) were placed on the base disc to filtrate the leaching water. This gravel filter layer was located at the bottom of the lysimeter to ensure an undisturbed water flow.

### Sample Preparation

Eight fruits and twelve leaves were randomly collected from the east, south, west, and north of the tree in mid-November every year of the field experiment. Four collected samples were mixed as one repetition. The collected samples were successively washed by a 0.1% aqueous solution of neutral detergent, 0.2% nitric acid solution, and deionized water for 30 s, respectively. The fruits were separated into pulp and peel, and then evenly into two parts after weighing. One part of the peel was used to measure the thickness, and the pulp was juiced and filtered. The filtrate was used for the assessment of total soluble solids (TSS), titratable acidity (TA) concentrations, the TSS/TA ratio, and the concentration of vitamin C (Vc). The leaf and a part of the peel and pulp samples were dried at 105°C in an oven for 30 min to inactivate enzymes, followed by drying at 60°C. Subsequently, the dried samples were smashed and passed through the 60-mesh sieves.

In the field experiment, soil samples were collected in mid-November every year. Three random soil collection points (near the drip line of the selected trees) were chosen per tree at depths of 0–20 cm, 20–40 cm, 40–60 cm. The samples were collected in the vertical direction of each soil extraction point of the selected trees. All the sampling tools were made of stainless steel to avoid any possible contamination. To prevent rain and dust, soil samples from all four trees were mixed and 1 kg of soil was taken to a clean indoor ventilation hood for air drying. After that, soil samples were ground and passed through the 20- and 100-mesh sieves.

The 30 L polyethylene container was used to collect the leachate in the lysimeter. When leachate samples reached a volume of 30 L, 200 ml of leachate samples were collected in polyethylene bottles and stored at 4°C until analysis. The first to eighth samples were collected on June 26, June 30, July 4, July 6, July 11, July 20, August 13, and September 10, respectively.

### Analysis of Fruit Quality

The analysis of fruit quality was based on the test method of citrus fresh fruit (GB/T8210-2011, China). Briefly, the fruit weight (FW) was weighed by an electronic balance with an accuracy of 0.01 g. The percentage yield of the juice (JY) was calculated from the volume of the obtained juice. The peel thickness (PT) was measured with a vernier caliper. The titratable acidity (TA) was measured by the standard base titration, and the digital sugar meter (ATAGO PAL-BX/ACID1) was used to analyze the total soluble solids (TSS). The TSS/TA ratio (TSA) is a ratio between the total soluble solids and the titratable acidity. Finally, the content of vitamin C (Vc) was analyzed by the 2, 6-dichlorophenol titration method (Ruck, 1963).

### Analysis of the Content of Nutrients in the Plant, Soil, and Leachate Sample

Plant analysis: Analyzed for the content of N, P, K, Ca, Mg, S, Cl according to the methods described by Bao (2000).

Soil analysis: The content of Cl was determined using an automatic potentiometric titrator (ZDJ-3D, China) by silver ion-titration. The content of soil avail-S in phosphate-acetic acid extraction was analyzed by ICP-OES (5110VDV, USA).

Leaching: The concentration of Cl was determined using the silver ion-titration method on an automatic potentiometric titrator (ZDJ-3D, China). A flame photometer (AP1200, China) was used for the determination of K concentration. The concentration of Ca and Mg in the samples digested with HNO_3_-HClO_4_ was determined by the atomic absorption spectrophotometry (Z2000, HITACHI, Japan). UV-Spectrophotometric was used for the determination of N-NO_3_ (UV-5200, China). The evaluation of ${\text{SO}}_{4}^{2-}$ was conducted with the Barium Sulfate Turbidimetry (UV-5200, China).

### Chlorine Leaching Factor Calculations


$$\begin{array}{l} \text{Cl leaching factor }\left(\text{ClLF},\%\right)=\frac{\left({\text{C}}_{\text{T}}-{\text{C}}_{\text{CK}}\right)*{\text{V}}_{1}*15000}{\text{C}{\text{l}}_{\text{r}}}\times 100\% \end{array}$$

where C_T_ and C_CK_ (mg L^−1^) are the concentrations of chlorine in Cl treated and 0 Cl plots, respectively, V_l_ (L) represents the volume of leached water, 15,000 is a conversion coefficient representing the planting density in soil core lysimeter experiment, and Cl_r_ is the amount of Cl applied rate (kg ha^−1^).

### Statistical Analysis

Analysis of variance (ANOVA) and single-factor analyses used Duncan multiple comparisons (*p* < 0.05) with the SPSS 22.0 software. The fruit quality index was plotted with TBtools. The relationship between Cl application rate and the yield of sweet orange was quantified by one-way regression analysis using Origin 2021 software. The correlations matrix (*p* < 0.05) between leaf nutrients and fruit quality was calculated and plotted by R software.

## Results

### Effects of Cl on Sweet Orange Yield

**Figure 1:**
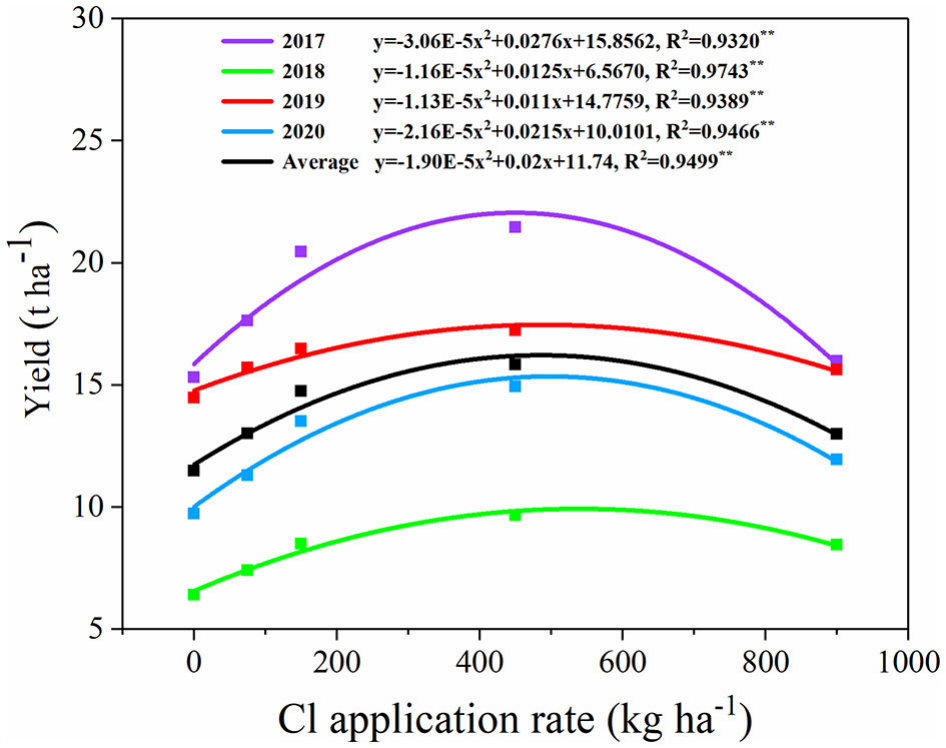
Regressions between annual Cl application rate and yield of sweet orange in the field experiment for 2017–2020.

Chlorine-containing fertilizer and their application rates significantly affected the yield of sweet orange during the growing years with an average increase of 13.2–37.9% compared with 0 kg ha^−1^ (Figure 1, *p* < 0.05). Among these, the 450 kg ha^−1^ treatment showed the highest yield every year (21.46 t ha^−1^ for 2017, 9.68 t ha^−1^ for 2018, 17.23 t ha^−1^ for 2019, and 14.94 t ha^−1^ for 2020, respectively). The mean maximum yield with the Cl application rates was 485 kg ha^−1^ (Figure 1).

### Effects of Cl on Sweet Orange Fruit Quality

**Figure 2:**
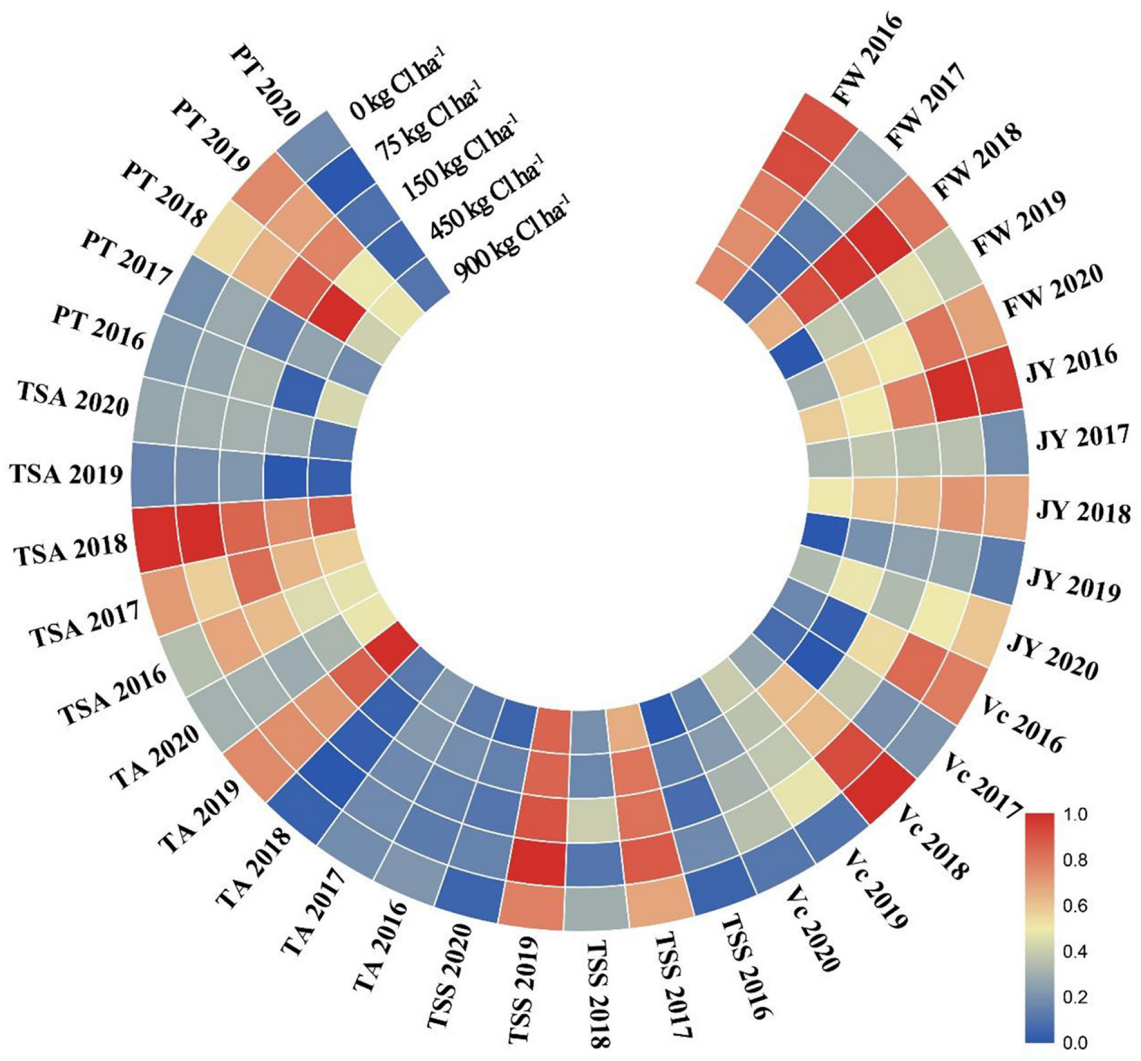
Effects of Cl-containing fertilizer on fruit weight (FW), juice yield (JY), Vc, TSS, TA, TSS/TA (TSA), and peel thickness (PT) of fruit quality in 5-year field experiment from 2016 to 2020. Bars are means of three replicates ± SD. Different letters (a, b, c) in each sub-figure represent significant differences at (*P* < 0.05). The data corresponding to this figure is shown in Supplementary Table S1.

The Cl-containing fertilizer improved fruit quality, specifically by increasing FW, JY, TSS, TSA, Vc, and PT. Meanwhile, Cl-containing fertilizer also significantly elevated the TSS and PT in 2018, Vc in 2016 and 2017, JY in 2016, and FW in 2017 and 2020 (Figure 2; Supplementary Table S1, *p* < 0.05). The JY and FW were significantly associated with the treatments, the TA, TSA, and PT were significantly related to growth years (Supplementary Table S1, *p* < 0.05), and the TSS was not only significantly related to years' change but also to treatment, while Vc was related neither to treatment nor years' change (Supplementary Table S1, *p* < 0.05).

### The Content of Cl in the Leaf, Pulp, and Peel

**Figure 3:**
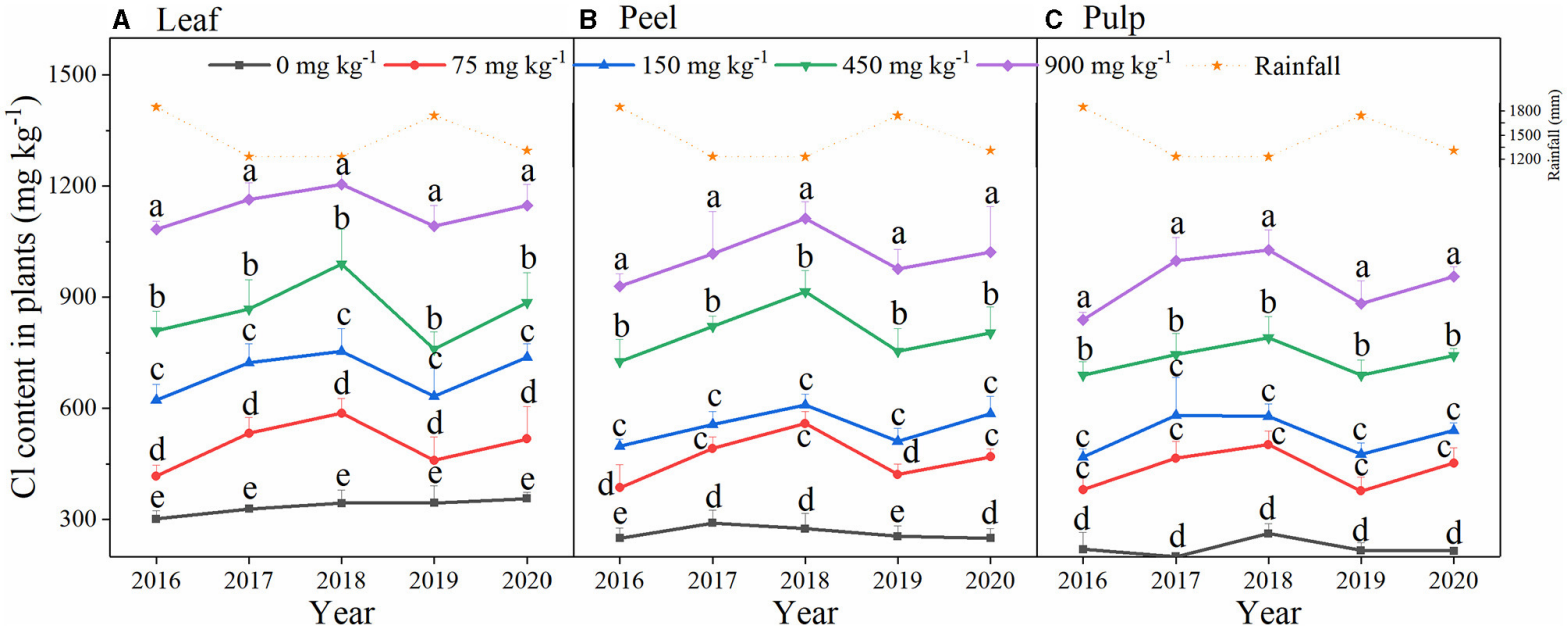
The content of chloride in leaf **(A)**, peel **(B)**, pulp **(C)** in a five-year field experiment from 2016 to 2020. Bars are means of three replicates ± SD. Different letters (a, b, c, d, e) in each sub-figure represent significant differences at (*P* < 0.05).

In the field experiment, the Cl-containing fertilizer significantly increased the content of Cl in the plants (Figure 3, *p* < 0.05). However, the leaf Cl content did not increase along with the year of application. There was a low content of Cl in the plant during 2016 and 2019, which appeared to result from the higher rainfall (1,849.5 and 1,742.1 mm) and the lower content of Cl in the soil. The highest Cl accumulation was observed in the treatment of 900 mg Cl ha^−1^ with the value of 1,205 mg kg^−1^ in the leaves (Figure 3A), 1,113 mg Cl ha^−1^ in the peel (Figure 3B), and 1,027 mg Cl ha^−1^ in the pulp (Figure 3C). The content of Cl was ranked as leaf > peel > pulp and leaf Cl was 16.62% higher than peel and was 28.88% higher than pulp (Figure 3).

### Nutrient Content in the Leaves

The Cl-containing fertilizer had a significant effect on the content of N, K, Ca, and S in the leaves (*p* < 0.05), but no significant effect on the content of P and Mg was noted. The content of N, K, Ca, and S in the leaves were not only significantly related to years' change, but also with the Cl input rate (Table 1). The content of N and K in the leaves significantly increased with the Cl input rate. The content of Ca in the treatment with KCl (150 kg Cl ha^−1^) was significantly lower than that in the treatment with K_2_SO_4_ (0 kg Cl ha^−1^), but extra Cl supplied by CaCl_2_, such as in the 450 kg Cl ha^−1^ and 900 kg Cl ha^−1^ treatments, could increase the content of Ca in the leaves (Table 1, *p* < 0.05). Furthermore, the average of S content was higher by 116.9 and 29.4% by application of 100% K_2_SO_4_ (0 kg Cl ha^−1^) and 50% K_2_SO_4_ (75 kg Cl ha^−1^) respectively, compared with no S-containing fertilizer treatment (the 150, 450, and 900 kg Cl ha^−1^ treatments), meanwhile, the annual average growth rate in the leaves was 30.6 and 18.5%.

**Table 1:** Data and statistical analysis of leaf nutrient content in 2016–2020.

**Year**	**Cl rate**	**N**	**P**	**K**	**Ca**	**Mg**	**S**
	**kg ha^**−1**^**	**g kg** ^**−1**^					
2016	0	28.3 ± 1.6b	1.7 ± 0.1a	19.3 ± 1.7a	38.5 ± 3.2a	3.1 ± 0.2a	2.6 ± 0.2a
	75	31.6 ± 1.6a	1.8 ± 0.1a	19.8 ± 0.9a	38.1 ± 3.4a	3.0 ± 0.2a	2.2 ± 0.1a
	150	30.4 ± 0.4ab	1.8 ± 0.1a	20.5 ± 0.7a	33.8 ± 4.9a	2.7 ± 0.1a	2.2 ± 0.2a
	450	29.3 ± 1.5ab	1.8 ± 0.1a	20.2 ± 1.1a	38.5 ± 2.5a	3.0 ± 0.4a	2.1 ± 0.3a
	900	31.07 ± 0.6a	1.8 ± 0.1a	20.6 ± 0.8a	40.3 ± 4.3a	3.2 ± 0.4a	2.2 ± 0.5a
2017	0	27.0 ± 1.0b	1.5 ± 0.0ab	23.2 ± 2.6a	29.2 ± 3.7ab	2.3 ± 0.3a	3.4 ± 0.1a
	75	28.3 ± 0.4ab	1.4 ± 0.1b	24.6 ± 2.9a	27.3 ± 1.1b	2.2 ± 0.1a	2.4 ± 0.1b
	150	27.3 ± 1.2b	1.4 ± 0.0bc	27.1 ± 0.5a	26.8 ± 1.4b	2.0 ± 0.4a	2.2 ± 0.1b
	450	29.2 ± 0.3a	1.5 ± 0.0ab	25.5 ± 3.8a	31.4 ± 3.0ab	2.1 ± 0.4a	2.1 ± 0.1b
	900	27.7 ± 0.6ab	1.5 ± 0.1a	28.3 ± 2.3a	34.0 ± 3.4a	2.3 ± 0.4a	2.2 ± 0.1b
2018	0	23.9 ± 1.0c	1.4 ± 0.1a	17.5 ± 0.9b	34.0 ± 0.4ab	2.1 ± 0.1a	4.8 ± 0.8a
	75	26.4 ± 0.6ab	1.5 ± 0.1a	19.8 ± 1.3a	33.4 ± 2.4ab	2.1 ± 0.3a	2.4 ± 03b
	150	25.5 ± 1.1bc	1.5 ± 0.1a	19.6 ± 0.2a	29.8 ± 2.6b	1.9 ± 0.3a	1.8 ± 0.2b
	450	25.8 ± 0.7bc	1.5 ± 0.1a	19.3 ± 1.0a	33.6 ± 1.9ab	2.1 ± 0.2a	2.0 ± 0.5b
	900	28.1 ± 1.8a	1.4 ± 0.2a	19.6 ± 0.8a	35.0 ± 3.7a	1.9 ± 0.2a	1.7 ± 0.3b
2019	0	24.1 ± 1.4b	1.5 ± 0.2a	18.0 ± 0.4b	37.2 ± 3.3a	2.0 ± 0.1a	5.8 ± 0.6a
	75	25.6 ± 1.0ab	1.4 ± 0.1a	18.9 ± 0.8ab	36.9 ± 5.2a	2.1 ± 0.4a	3.0 ± 0.3b
	150	27.0 ± 1.0a	1.4 ± 0.1a	18.5 ± 1.0ab	35.4 ± 4.0a	2.0 ± 0.3a	2.5 ± 0.1b
	450	25.6 ± 0.8ab	1.4 ± 0.1a	19.5 ± 0.8a	37.8 ± 5.1a	1.9 ± 0.2a	2.2 ± 0.2b
	900	26.9 ± 1.5a	1.4 ± 0.1a	19.6 ± 0.7a	39.6 ± 4.2a	1.9 ± 0.1a	2.2 ± 0.2b
2020	0	26.4 ± 0.8b	1.4 ± 0.1a	18.4 ± 0.8b	34.9 ± 0.2a	2.0 ± 0.4a	7.4 ± 0.5a
	75	30.0 ± 0.6ab	1.4 ± 0.1a	18.8 ± 0.8ab	34.0 ± 0.8ab	2.0 ± 0.6a	4.2 ± 0.1b
	150	30.4 ± 3.6ab	1.4 ± 0.1a	19.7 ± 0.4ab	30.97 ± 3.1b	1.9 ± 0.1a	2.9 ± 0.3c
	450	30.0 ± 1.2ab	1.4 ± 0.1a	20.1 ± 0.2a	34.0 ± 0.9ab	1.9 ± 0.1a	2.5 ± 0.3c
	900	31.6 ± 4.0a	1.4 ± 0.1a	19.3 ± 1.2ab	35.5 ± 2.0a	1.9 ± 0.2a	2.4 ± 0.1c
**F test**	Rate (R)	8.7^***^	0.4^ns^	4.8^**^	5.3^***^	1.2^ns^	174.4^***^
	Year (Y)	26.0^***^	53.3^***^	55.4^***^	16.2^***^	36.1^***^	62.1^***^
	R^*^Y	1.0^ns^	1.3^ns^	0.8^ns^	0.3^ns^	0.4^ns^	17.1^***^

*Values are means of three replicates. Values represent means ± SE (n = 3)*.

* *p < 0.05*,

** *p < 0.01, and*

*** *p < 0.001*.

*Different letters in each sub-figure represent significant differences at (P < 0.05)*.

### Correlations Between Fruit Quality and Mineral Nutrients in the Leaves

**Figure 4:**
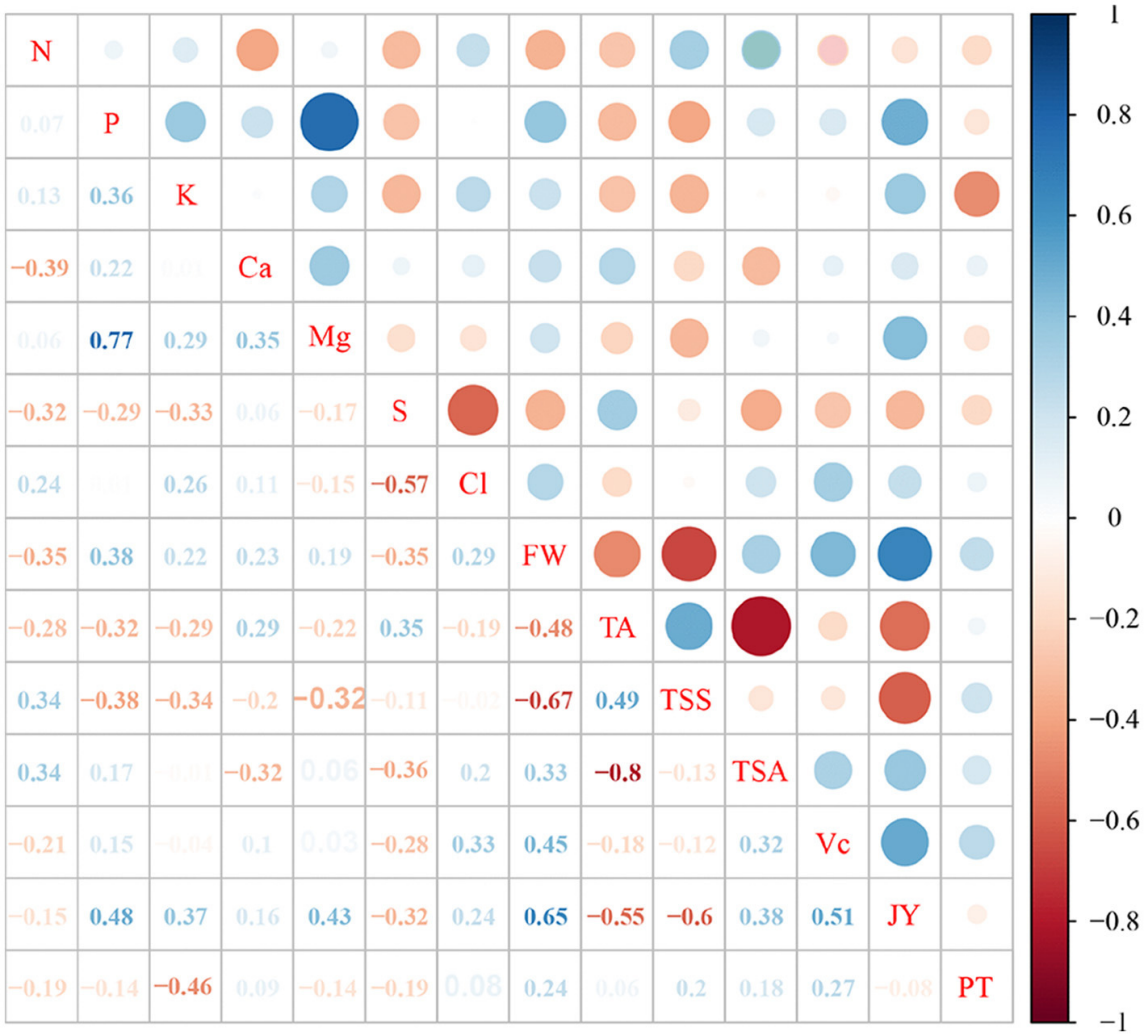
Correlation analyses of leaf mineral nutrients and fruit quality. N, P, K, Ca, Mg, S, and Cl represent the content of N, P, K, Ca, Mg, S, and Cl in the levaes; FW, fruit weight; TA, titratable acid; TSS, total soluble solid; TSA, TSS/TA; Vc, vitamin C; JY, juice yield; PT, peel thickness.

The FW had significantly positive correlations with the content of P, Ca, and Cl in the leaves, but negative correlations with the content of N and S (Figure 4, *p* < 0.05). There were dramatically positive correlations was found between the TA and the Ca and S in the leaves, but negative correlations with N, P, and K (*p* < 0.05). The TSS only had significantly positive correlations with N and showed negative correlations with P, K, and Mg (*p* < 0.05). The content of Ca and S was found to be negatively correlated with the TSA, which was positively correlated with N (*p* < 0.05). The Vc possessed significantly positive correlations with Cl content and negative correlations with S in the leaves (*p* < 0.05). Similarly, there were remarkable positive correlations between JY and P, K, Mg, and Cl content in the leaves, but negative correlations with S (*p* < 0.05). The PT was prominently negatively correlated with S in the leaves (Figure 3; *p* < 0.05). Interestingly, the content of N and K had significantly positive correlations with Cl in the leaves, but negative correlations with the content of S in the leaves.

### The Content of Cl and Avail-S in Soil

**Figure 5:**
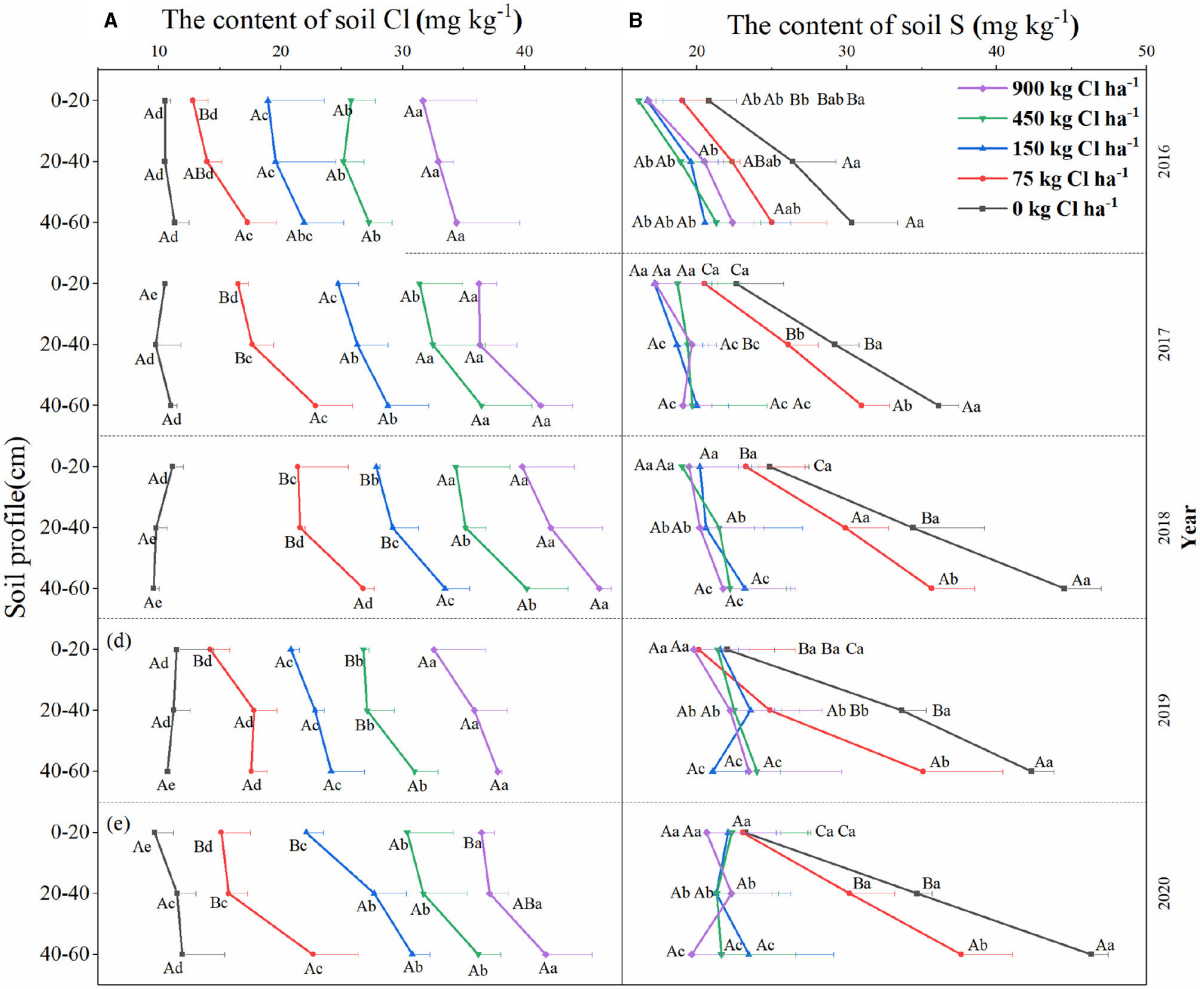
The content of Cl **(A)** and S **(B)** changes in 0–60 cm soil layer in a 5-year field experiment from 2016 to 2020. Bars are means of three replicates ± SD. The different lowercase letters (a, b, c, d, e) indicate significant differences between the Cl treatment at (*P* < 0.05). The different uppercase letters (A, B, C) indicate significant differences between the soil layer at (*P* < 0.05).

The Cl-containing and S-containing fertilizer significantly increased the content of Cl and avail-S in the soil (Figure 5, *p* < 0.05). The content of Cl in the soil (0–60 cm) increased significantly as soil depth increased. Specifically, the Cl content in the 40–60 cm soil layer was 21.15 and 14.77% higher than that in the 0–20 and 20–40 cm soil layers, respectively (except for the 0 kg Cl ha^−1^ treatment). Similarly, the content of avail-S in the 40–60 cm soil layer was 65.1 and 24.3% higher than the 0–20 and 20–40 cm layers, respectively with the application of S-containing fertilizer. It can be seen that the content of Cl in the soil in each treatment did not increase with the year of Cl-containing fertilizer, whereas the content of soil avail-S with 2020 had 16.6, 33.3, and 51.7% higher than 2016 in 0–20, 20–40, and 40–60 cm soil layer (except for 150, 450, and 900 kg Cl ha^−1^). Interestingly, the Cl accumulation in soil may have been affected by rainfall as the content of Cl in the soil was low in 2016 and 2019, which were 2 years with high rainfall (1,849.5 and 1,742.1 mm, respectively). Simultaneously, the dynamic changes in the content of Cl at the 0–60 cm soil layer during 2016–2018 were analyzed (Supplementary Figure S2). The variation of soil Cl content showed inverted U-shaped curves. The soil Cl content was ranked as September > December > June, with September being 34.15 and 12.98% higher than June and December, respectively (except 0 kg Cl ha^−1^).

### Anions and Cations in the Leachate

**Figure 6:**
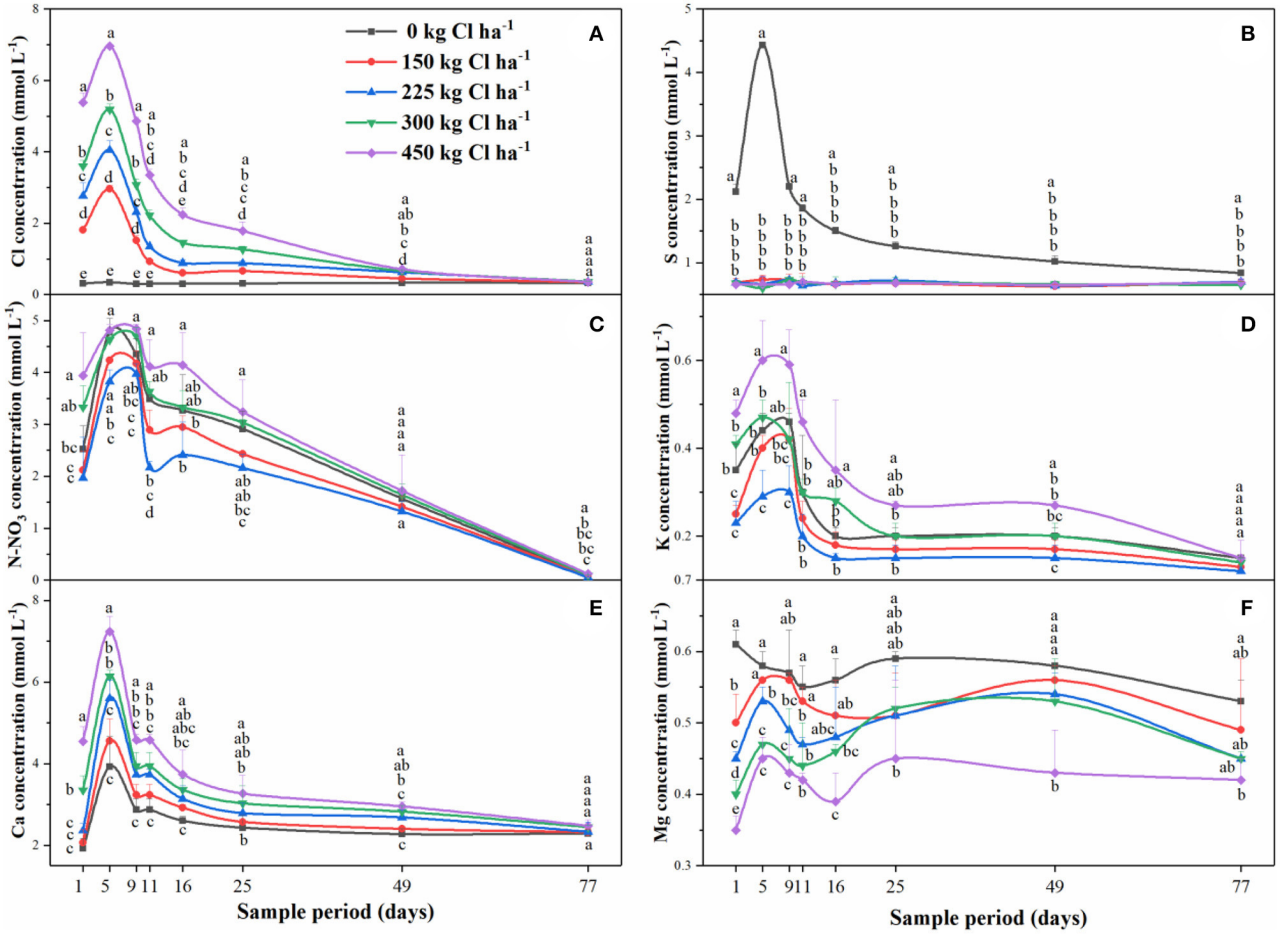
The concentration of Cl **(A)**, S **(B)**, N-NO_3_ **(C)**, K **(D)**, Ca **(E)**, and Mg **(F)** dynamics changes in leachate by application of Cl-containing fertilizers on sweet orange. Bars are means of three replicates ± SD. Different letters (a, b, c, d, e) in each sub-figure represent significant differences at (*P* < 0.05).

There were a total of eight leaching events during the soil core lysimeter experiment (Figure 6). The leachate with Cl-containing fertilizer had 3–11 folds more of average chloride concentrations than non-chlorine treatment, and the average Cl leaching factor was 77.0% in the soil core lysimeter experiment (Figure 6A). The concentration of ${\text{SO}}_{4}^{2-}$ in leachate increased remarkably by use of the K_2_SO_4_ fertilizer, but no effect was observed in the treatments of Cl (Figure 6B). Furthermore, the concentration of N-NO_3_ and K^+^ treated with K_2_SO_4_ was higher than in the KCl treatment, the highest content was observed in the treatment of 450 kg Cl ha^−1^. The Ca^2+^ concentration increased with the Cl application rate, but Mg^2+^ concentration reduced.

## Discussion

### Application of Cl-Containing Fertilizer on Citrus Is Feasible Under the Premise of Rainfall Higher Than Evaporation

In field trials, we can only observe the dynamic changes of the Cl content in the soil, it is difficult to obtain the soil leaching solution. The soil core lysimeter solved the difficult collection of leachates and can better evaluate the pattern of ion migration (Derby et al., 2002; Di and Cameron, 2005; Zhao et al., 2010).

Chlorine mainly exists in the soil in the form of chloride and was usually considered as a tracer to measure soil water movement (Geilfus, 2018a). When the rainfall exceeds the evaporation, there is a downward movement of Cl. However, the rainfall may lead to the opposite trend of Cl, namely upward movement, resulting in an increasing content of Cl in the soil. Water fluxes are the key factors of Cl migration in soil (Havlin et al., 1985). In our research, despite the Cl content in soil being increased remarkably by Cl-containing fertilizer and soil depth (0–60 cm), the Cl content in soil did not increase with years of consecutive application over 5 years of such application with an average rainfall 1,475 mm in the field experiment (Figure 5A). The high rainfall resulted in the rapid dissolution of Cl-containing fertilizer, accompanied by diffusion and leaching. These processes mainly occur in the rainy season from March to August. Within a year, the Cl dissolution rate was higher than the leaching rate during the period from June to September, while the opposite result was observed from September to December, which helps explain the reason for the highest soil Cl content in September (Supplementary Figure S2). As explained above, Cl-containing fertilizer underwent a dissolution-diffusion-leaching process in the soil. Similarly, the fact that the ion concentration first rose and then decreased in leachate further supports this process (Figure 6). The content of Cl in the soil is affected by rainfall and evaporation (Burns, 1974). Herein, the further research result revealed that the Cl leaching factor reached 77.0% in the soil core lysimeter experiment (Figure 6A). In most southern areas of the Yangtze River, the annual rainfall is over 1,000 mm, and the accumulation of Cl in the soil is generally <10%, which is consistent with our research (Mao et al., 2001), illustrating that chloride is easy to migrate since it is not easily adsorbed by soil.

The kinetics of ions is associated with the charge balance in the soil, which in turn depends on the process of ion exchange, nutrient loss caused by soil leaching, and acidification (Souza et al., 2006). The release of K^+^, Ca^2+^, and Mg^2+^, etc cations increased, and leached loss more easily in acid soil (Quaggio, 2000) with Cl, N-NO_3_, and S as the main accompanying anions (Li et al., 2015). The result suggested that the concentration of Cl, N-NO_3_, Ca^2+^, and S were much higher than K^+^ and Mg^2+^ concentrations in the leachate (Figure 6). The Cl and Ca^2+^ concentration of the leachate conspicuously increased after the application of Cl-containing fertilizer, indicating the contribution rate of Ca^2+^ is greater than K^+^ and Mg^2+^, etc cations in Cl leaching processes.

In general, soil avail-S mainly exist as ${\text{SO}}_{4}^{2-}$ in the soil, and the different cation migration may result from the low solubility of CaSO_4_ resulting in the slow migration of ${\text{SO}}_{4}^{2-}$ h in the soil compared to Cl. Iron and aluminum oxides had a strong absorption capacity for ${\text{SO}}_{4}^{2-}$ in soil, and this absorption capacity was further enhanced in acid soil (Bolan et al., 2003). In the current study, the content of soil avail-S was enhanced yearly by the long-term application of K_2_SO_4_ (Figure 5B). And the concentration of Ca^2+^ in the K_2_SO_4_ treatment was lower than that of the KCl treatment in leachate (Figure 6E). Likewise, about 24.6% of S was adsorbed by soil and reached the maximum at 45–60 cm soil layer, after consecutive application of S-containing fertilizer over 6 years (Saha et al., 2001). Furthermore, Cowling et al. (1992) reported that the reoxidation of metal sulfides will produce hydrogen ions, which would induce soil acidification. Gradually, the long-term application of S-containing fertilizer will cause avail-S to accumulate in soil and lead to more severe soil acidification.

The soil is considered not only one of the important sources of plant nutrition, but also the main reservoir and a source of Cl, which directly affects the uptake of Cl by plants. This 5-year field experiment showed the relatively stable content of Cl in the leaves, peel, and pulp over the 5 years, while S content was scaling up. The Cl content in the leaves of olives and mandarin, which have been using saline irrigation water for a long time, have also not increased continuously, which is consistent with the present results (Melgar et al., 2009; Nicolás et al., 2016). The reasons may be due to (i) Cl is used as the nutritient for plant growth; (ii) a part of Cl was taken away by the fruit; (iii) since most of the Cl is being leached to a deeper soil layer, that reduced the amount of Cl uptake by citrus. Conversely, the content of S in the leaves increased yearly by the application of S-containing fertilizer during 2016–2020, which may be the result of the continuous accumulation of soil avail-S therefore promoting the absorption by citrus (Table 1). Excessive S application could improve the availability of heavy metals in soil, meanwhile, promoting the absorption and accumulation of metal sulfide by plants (Zakari et al., 2021), implying that the difference in adsorption capacity of Cl and S by soil resulted in the divergent accumulation of Cl and S in the leaves.

To sum up, in acidic soil, metal cations are released more easily causing more Cl leaching, in which calcium is the main accompanying ion. Therefore, only a small amount of chlorine is absorbed by citrus. However, sulfate will form complexes with metal cations (e.g., Ca, Fe, Al, etc.) and cause deposition in the soil and lead to the increase of S absorption by citrus, and produce hydrogen ions resulting in serious acidification of the soil.

### Appropriate Application of Cl-Containing Fertilizer Improved Citrus Yield and Fruit Quality

The initiative uptake of Cl by plants was far more than 200–400 mg kg^−1^. A remarkable improvement of growth and biomass was observed when Cl existing in plants was higher than micronutrient levels (Chen et al., 2010; Franco-Navarro et al., 2016). Through the determination of leaf samples from 670 species of terrestrial plants belonging to 138 families, it was shown that the highest Cl content is about 0.5% (Watanabe et al., 2007; Colmenero et al., 2019). In the present study, the Cl content in the leaves ranging from 335 to 1,138 mg kg^−1^ showed no symptoms of Cl stress and harvested the highest yield at the 485 kg Cl ha^−1^ input rate. The long-term application of KCl increased the yield of sweet orange and lemon in Brazil (Quaggio et al., 2002, 2006, 2011), which is consistent with our conclusion that Cl improved the photosynthetic activity, leaf cell division rates, water use efficiency, and biomass promoting the growth of citrus (Brumos et al., 2010; Franco-Navarro et al., 2019). Meanwhile, the accumulation of Cl subsided the N-NO_3_ accumulation efficiency in plants, thereby improving nitrogen use efficiency (Rosales et al., 2020), similar results were obtained in ${\text{PO}}_{4}^{3-}$ and ${\text{SO}}_{4}^{2-}$ (Franco-Navarro et al., 2016). Collectively, the Cl content in plants is at a level equivalent to the macronutrient level (302–1,205 mg Cl kg^−1^) can promote the growth of citrus and improve its yield.

It is still unclear whether the beneficial effect on plants is a result of the direct effect of chloride or the effect of concomitant ions (Flowers, 1988). Despite the fact that the application of Cl-containing fertilizer had no significant effect on fruit quality during 2016–2020, the application of Cl-containing fertilizer did nonetheless cause a slight improvement in fruit quality (Figure 2). The correlation between leaf mineral nutrition and fruit quality analysis showed that leaf Cl is positively correlated with the FW, Vc, and JY of sweet orange. Appropriate Cl input conspicuously increased the FW whereas N input rate showed a negative effect on the FW (Quaggio et al., 2002), which agrees with our research. Improving the absorption of Cl extremely increased Vc in the fruit of strawberry (Xu et al., 1999). The JY content of sweet orange increased with the KCl input rate (Quaggio et al., 2011), revealing that Cl improved fruit quality based on enhancing the FW, Vc, and JY of citrus.

Widely, N and K are the important elements for citrus yield and fruit quality (Quaggio et al., 2002). Here, it was found that Cl content in the leaves is positively correlated with the contents of N and K (Figure 4). The content of N and K is greatly higher under the KCl treatment in comparison to the K_2_SO_4_ treatment (Table 1). Chlorine has the tendency of increasing K content in the leaves of citrus seedlings (Xu et al., 1999). Meanwhile, previous reports showed that Cl had no significant effect on the content of N in the leaves, in turn, Cl has decreased N-NO_3_ accumulation in the vacuole, promote its assimilation, which improves the utilization of N and plant growth (Rosales et al., 2020). The leaf N content was positively correlated with TSS, while it was passively correlated with TA. He et al. (2003) reported the same correlation between leaf N and TA, TSS in grapefruit, confirming that N reduced TA and increased TSS to improve the citrus flavor. Furthermore, the content of K in the leaves was noticeably positively correlated with JY. The JY content has significantly increased by application of K fertilizer to improve leaf K content in Kinnow (Ashraf et al., 2010), indicating that K improved the internal fruit quality. Therefore, supplying Cl increased the leaf N and K content, which has improved the citrus flavor and JY.

## Conclusion

The present results showed that Cl experienced a process of dissolution-diffusion-leaching in the soil after the application of Cl-containing fertilizer. Even though the content of Cl in soil elevated with Cl input rate, it did not increase yearly in a 5-year field experiment. Little Cl uptake by citrus and no year-on-year increase might be a result of the leaching of the majority Cl to the deeper soil layers. Simultaneously, Ca^2+^ is the accompanying cation of Cl during its leaching. Chlorine enhanced the yield and FW of sweet orange, and, improved the flavor and JY by promoting the absorption of N and K in the leaves. Furthermore, the content of S in soil and leaf has increased year after year from the long-term application of K_2_SO_4_ due to ${\text{SO}}_{4}^{-}$ being easily complexed with metal cations, resulting in accumulation and accelerated soil acidification that may be adverse for citrus production. Therefore, it was recommended that the application of Cl-containing fertilizer could improve citrus yield and fruit quality, and avoid the risk of excessive S in citrus production.

## Data Availability Statement

The original contributions presented in the study are included in the article/Supplementary Material, further inquiries can be directed to the corresponding author.

## Author Contributions

QT and XDL designed and supervised this study. XDL conducted the experiments, performed data interpretation, and drafted the manuscript. ZZ and ZT helped in interpreting the results of the study. YL, XML, and ZD helped in the experiment and determining nutrition concentration. CH, SW, and MR helped to revise the manuscript grammatically. All authors read and approved the final manuscript.

## Funding

This work was supported by the National Key Research and Development Program of China (2019YFD1001400), the Modern Citrus Industry Technology System of China (CARS-26), and the National Key Research and Development Program of China (2019YFD1000103).

## Conflict of Interest

The authors declare that the research was conducted in the absence of any commercial or financial relationships that could be construed as a potential conflict of interest.

## Publisher's Note

All claims expressed in this article are solely those of the authors and do not necessarily represent those of their affiliated organizations, or those of the publisher, the editors and the reviewers. Any product that may be evaluated in this article, or claim that may be made by its manufacturer, is not guaranteed or endorsed by the publisher.
