# Supplementary material for: Migration of Chlorine in Plant–Soil–Leaching System and Its Effects on the Yield and Fruit Quality of Sweet Orange
Source: Front Plant Sci. 2021 Oct 11;12:744843. doi: 10.3389/fpls.2021.744843 (PMC8542884; doi:10.3389/fpls.2021.744843)
Supplement: Supplementary file 1 [file Table_1.docx]

**Table S1** Data and statistical analysis of fruit quality index in 2016-2020

| Year | Cl rate | TSS | TA | TSA | Vc | JY | FW | PT |
| --- | --- | --- | --- | --- | --- | --- | --- | --- |
|  |  | % | % |  | mg/100g | % | g | mm |
| 2016 | 0 | 9.94±0.31a | 0.53±0.06a | 19.01±2.25a | 28.45±2.45bc | 63.65±0.75b | 293.75±51.55a | 4.91±0.75a |
|  | 75 | 10.40±0.73a | 0.56±0.06a | 18.73±2.87a | 27.09±1.43c | 62.39±4.44b | 292.50±15.91a | 4.01±0.33a |
|  | 150 | 10.20±0.18a | 0.54±0.08a | 20.91±5.97a | 32.11±0.55ab | 67.04±3.23ab | 297.50±14.14a | 4.65±0.25a |
|  | 450 | 10.54±0.94a | 0.53±0.08a | 21.69±5.11a | 35.06±2.21a | 70.94±0.53a | 311.88±34.47a | 4.53±0.72a |
|  | 900 | 10.10±1.33a | 0.58±0.04a | 17.43±1.25a | 34.58±3.03a | 70.53±1.85a | 309.86±37.81a | 4.43±0.25a |
| 2017 | 0 | 12.20±0.31a | 0.59±0.01a | 20.24±0.90a | 27.51±1.67b | 59.37±0.35a | 218.76±0.88b | 4.33±0.35a |
|  | 75 | 12.70±1.03a | 0.58±0.04a | 21.16±3.12a | 26.82±0.50b | 60.22±3.53a | 219.17±6.07b | 4.52±0.15a |
|  | 150 | 12.74±0.62a | 0.55±0.08a | 23.62±4.20a | 30.64±1.44a | 59.87±2.32a | 225.00±2.50b | 4.22±0.11a |
|  | 450 | 12.95±0.50a | 0.56±0.04a | 20.31±5.12a | 28.75±0.43ab | 59.96±2.44a | 244.07±3.09a | 4.56±0.54a |
|  | 900 | 12.27±0.09a | 0.56±0.04a | 22.07±1.62a | 28.87±1.05ab | 56.97±5.69a | 241.88±2.65a | 4.34±0.34a |
| 2018 | 0 | 10.60±0.20b | 0.52±0.01a | 24.23±1.63a | 29.46±1.44a | 62.38±1.31a | 283.12±1.61a | 4.85±0.60c |
|  | 75 | 10.50±0.09ab | 0.48±0.03a | 22.46±1.91a | 32.93±0.50a | 64.01±13.04a | 310.42±5.71a | 6.14±0.19a |
|  | 150 | 11.32±0.31ab | 0.48±0.04a | 23.90±2.79a | 32.98±0.65a | 64.64±4.39a | 318.02±8.16a | 5.88±0.56ab |
|  | 450 | 10.30±0.14ab | 0.46±0.09a | 25.84±0.08a | 35.86±4.72a | 66.17±0.98a | 320.17±4.23a | 5.37±0.40abc |
|  | 900 | 10.96±0.86a | 0.48±0.07a | 25.8±0.07a | 36.64±5.83a | 65.41±1.68a | 299.45±8.71a | 5.13±0.65bc |
| 2019 | 0 | 12.85±0.33a | 0.10±0.26a | 13.17±3.36a | 30.77±2.17a | 53.78±5.06a | 211.92±10.87a | 5.01±0.53a |
|  | 75 | 12.85±0.57a | 0.93±0.05a | 12.83±.17a | 30.41±5.15a | 57.14±3.75a | 256.31±7.61a | 5.03±0.58a |
|  | 150 | 13.02±0.02a | 0.85±0.15a | 15.66±2.57a | 30.58±1.46a | 58.12±1.86a | 248.87±9.80a | 5.63±0.41a |
|  | 450 | 13.35±0.07a | 0.86±0.10a | 15.23±1.66a | 31.54±1.77a | 58.47±4.55a | 262.96±6.81a | 5.47±0.42a |
|  | 900 | 12.60±0.46a | 0.86±0.14a | 14.83±2.17a | 27.79±1.04a | 55.94±1.60a | 253.83±8.90a | 5.61±0.08a |
| 2020 | 0 | 10.10 ±0.30a | 0.72±0.10a | 14.08±1.68a | 28.37±2.24a | 59.67±1.23a | 244.53±2.15b | 4.16±0.46a |
|  | 75 | 10.43±0.21a | 0.64±0.09a | 16.58±2.64a | 29.09±1.78a | 62.18±2.55a | 273.46±6.54ab | 4.05±0.43a |
|  | 150 | 10.30±0.35a | 0.62±0.07a | 16.81±2.25a | 29.95±3.01a | 59.61±1.49a | 266.66±6.75ab | 4.13±0.20a |
|  | 450 | 10.47±0.21a | 0.63±0.04a | 16.74±0.72a | 30.34±0.83a | 62.40±3.08a | 299.52±5.80a | 3.94±0.98a |
|  | 900 | 10.10±0.72a | 0.63±0.10a | 16.36±2.45a | 27.89±0.78a | 64.05±1.08a | 286.66±2.88a | 4.33±1.11a |
| **F test** | Rate (R) | 4.91^*^ | 1.9^ns^ | 1.0^ns^ | 1.2^ns^ | 11122.1^***^ | 17.1^***^ | 2.0^ns^ |
|  | Year (Y) | 178.7^***^ | 22.5^***^ | 21.9^***^ | 3.5^ns^ | 0.79^ns^ | 0.8^ns^ | 12.6^***^ |
|  | R*Y | 3.24^*^ | 1.0^ns^ | 0.4^ns^ | 0.7^ns^ | 8.2^**^ | 2.9^ns^ | 1.2^ns^ |

Values are means of three replicates. Values represent means ± SE (n = 3). * significant at p < 0.05. ** significant at p < 0.01. *** significant at p < 0.001. Different letters in each sub-figure represent significant differences at (P < 0.05).
