# Supplementary figures and images for: Migration of Chlorine in Plant–Soil–Leaching System and Its Effects on the Yield and Fruit Quality of Sweet Orange
Source: Front Plant Sci. 2021 Oct 11;12:744843. doi: 10.3389/fpls.2021.744843 (PMC8542884; doi:10.3389/fpls.2021.744843)

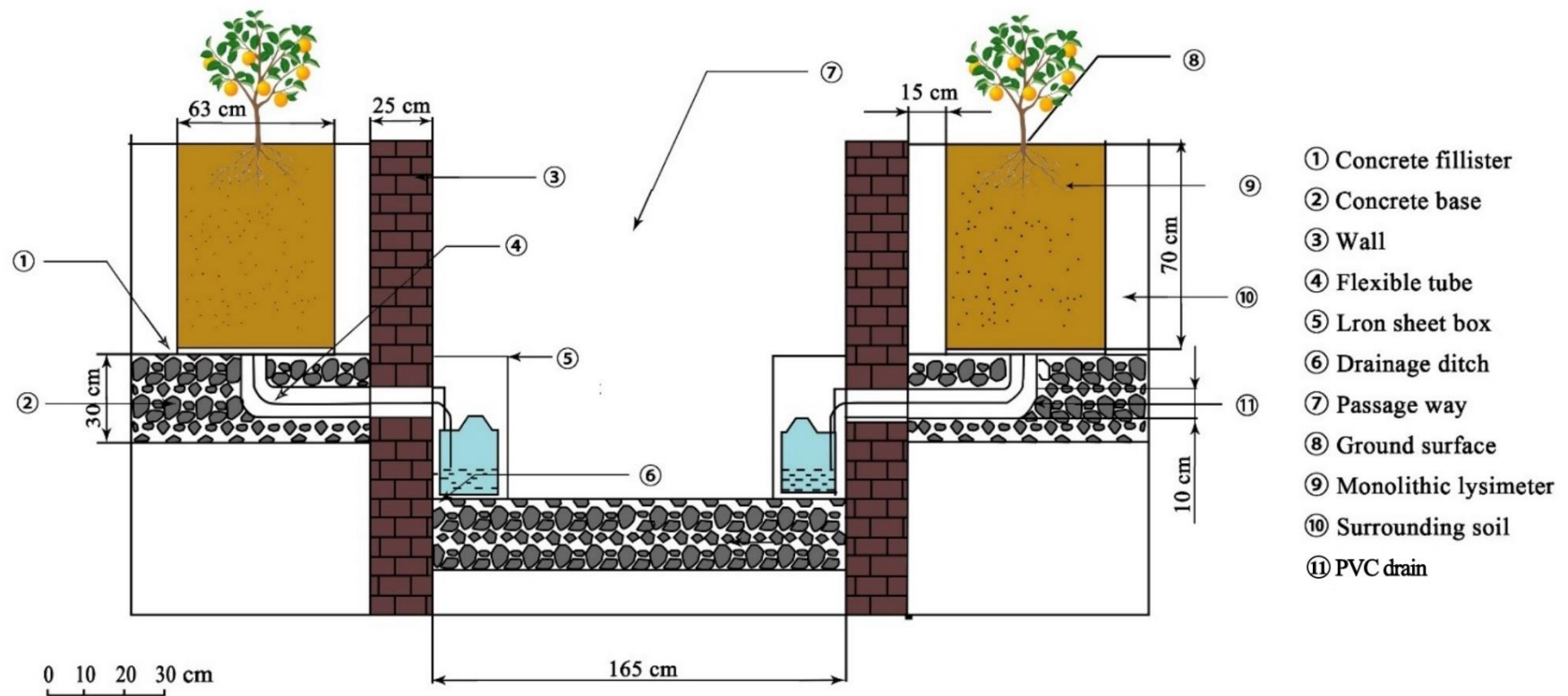

**Supplementary Figure S1** The vertical structure of the design for large soil core lysimeter.

Supplement: Supplementary file 2 [file Image_1.pdf]
