# Supplementary material for: Migration of Chlorine in Plant–Soil–Leaching System and Its Effects on the Yield and Fruit Quality of Sweet Orange
Source: Front Plant Sci. 2021 Oct 11;12:744843. doi: 10.3389/fpls.2021.744843 (PMC8542884; doi:10.3389/fpls.2021.744843)
Supplement: Supplementary file 3 [file Image_2.pdf]

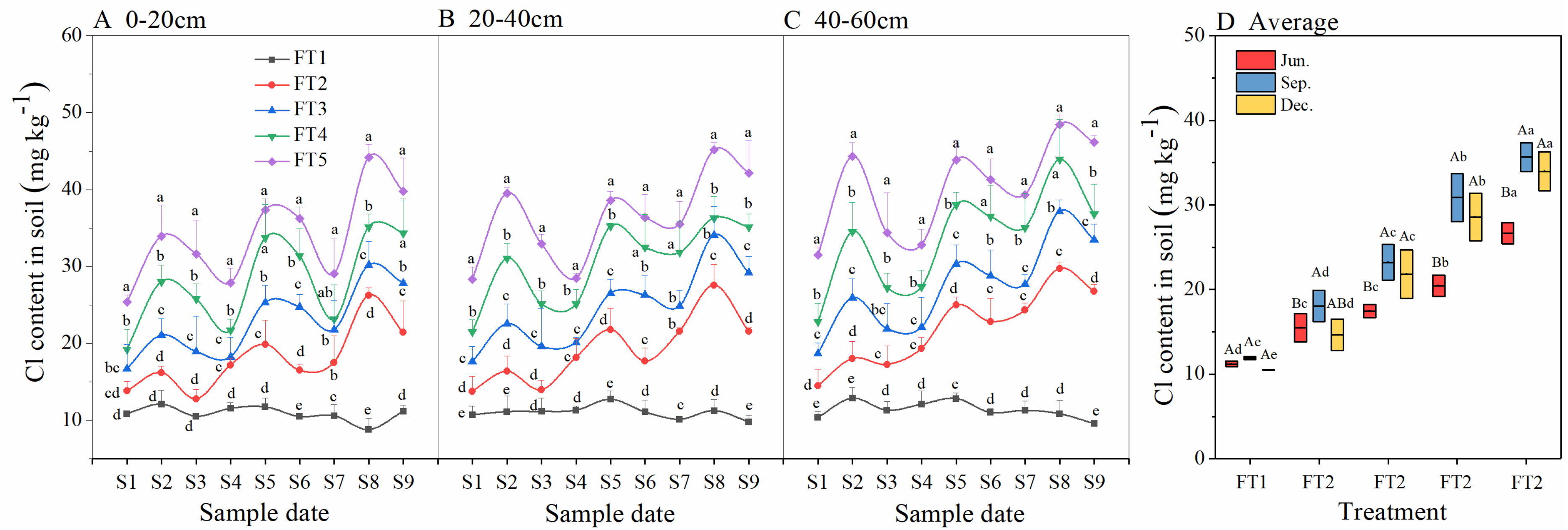

Supplementary Figure S2 Soil chloride dynamics change in the 0–20 cm (a), 20–40 cm (b) and 40–60 cm (c) soil layers, and chloride in the 0-60cm soil layers (d) at June, September and December under different chloride treatments in five-year field experiment from 2016-2018. S1: June 2016, S2: September 2016, S3: December 2016, S4: June 2017, S5: September 2017, S6: December 2017, S7: June 2018, S8: September 2018, S9: December 2018. Bars are means of three replicates ± SD. Different letters (a, b, c, d) in each sub-figure represent significant differences at ($P < 0.05$).
